# Impact of attaining aggressive vs. conservative PK/PD target on the clinical efficacy of beta-lactams for the treatment of Gram-negative infections in the critically ill patients: a systematic review and meta-analysis

**DOI:** 10.1186/s13054-024-04911-5

**Published:** 2024-04-16

**Authors:** Milo Gatti, Pier Giorgio Cojutti, Federico Pea

**Affiliations:** 1 Department of Medical and Surgical Sciences, Alma Mater Studiorum, University of Bologna, Bologna, Italy; 2 Clinical Pharmacology Unit, IRCCS Azienda Ospedaliero-Universitaria di Bologna, Via Massarenti 9, 40138 Bologna, Italy

**Keywords:** Critically ill patients, Beta-lactams, Aggressive PK/PD target attainment, Clinical efficacy, Risk score for failure in attaining aggressive PK/PD target

## Abstract

**Background:**

To perform a systematic review with meta-analysis with the dual intent of assessing the impact of attaining aggressive vs. conservative beta-lactams PK/PD target on the clinical efficacy for treating Gram-negative infections in critical patients, and of identifying predictive factors of failure in attaining aggressive PK/PD targets.

**Methods:**

Two authors independently searched PubMed-MEDLINE and Scopus database from inception to 23rd December 2023, to retrieve studies comparing the impact of attaining aggressive vs. conservative PK/PD targets on clinical efficacy of beta-lactams. Independent predictive factors of failure in attaining aggressive PK/PD targets were also assessed. Aggressive PK/PD target was considered a100%fT_>4xMIC_, and clinical cure rate was selected as primary outcome. Meta-analysis was performed by pooling odds ratios (ORs) extrapolated from studies providing adjustment for confounders using a random-effects model with inverse variance method.

**Results:**

A total of 20,364 articles were screened, and 21 observational studies were included in the meta-analysis (N = 4833; 2193 aggressive vs. 2640 conservative PK/PD target). Attaining aggressive PK/PD target was significantly associated with higher clinical cure rate (OR 1.69; 95% CI 1.15–2.49) and lower risk of beta-lactam resistance development (OR 0.06; 95% CI 0.01–0.29). Male gender, body mass index > 30 kg/m^2^, augmented renal clearance and MIC above the clinical breakpoint emerged as significant independent predictors of failure in attaining aggressive PK/PD targets, whereas prolonged/continuous infusion administration of beta-lactams resulted as protective factor. The risk of bias was moderate in 19 studies and severe in the other 2.

**Conclusions:**

Attaining aggressive beta-lactams PK/PD targets provided significant clinical benefits in critical patients. Our analysis could be useful to stratify patients at high-risk of failure in attaining aggressive PK/PD targets.

**Supplementary Information:**

The online version contains supplementary material available at 10.1186/s13054-024-04911-5.

## Background

Sepsis and septic shock are two leading causes of patient’s admission in the intensive care unit (ICU) worldwide, and may cause remarkable morbidity and mortality [1]. Beta-lactams are considered mainstay of empirical and targeted therapy of septic patients, since they adequately cover the vast majority of Gram-negative bacteria responsible for these cases [2, 3].

Beta-lactams are time-dependent antibiotics whose efficacy depends on the time that the free (*f*) concentrations are maintained above the minimum inhibitory concentration (MIC; T_>MIC_) of the pathogen [4]. A conservative PK/PD target of 40–100% *f*T_>MIC_ was traditionally considered as sufficient for granting clinical efficacy [4]. However, recent preclinical and clinical studies showed that aggressive PK/PD targets defined as 100% *f*T_>4x MIC_ were associated with better microbiological eradication rates and less propensity of resistance occurrence compared to the conservative ones [5–7].

Consequently, this is progressively leading to a paradigm shift in the theoretical principles for optimizing treatment with beta-lactams [8], even if this issue is still a matter of debate [9].

Attaining aggressive PK/PD target of beta-lactams was shown to be more probable when adopting prolonged/continuous infusion administration and/or a therapeutic drug monitoring (TDM)-guided dosing adaptative approach [10, 11]. However, regardless of applying this, failure rate in attaining aggressive PK/PD target of beta-lactams may still be remarkable in the critically ill patients due to pathophysiological features causing wide inter- and intra- individual pharmacokinetic variability [12].

Based on this, we performed a systematic review with meta-analysis with the dual intent of assessing the impact of attaining aggressive vs. conservative PK/PD target in the clinical efficacy of beta-lactams for the treatment of Gram-negative infections among the critically ill patients, and of identifying the patient’s conditions associated with failure in attaining aggressive PK/PD targets.

## Methods

A systematic review and meta-analysis was carried out for: (a) assessing the impact of attaining aggressive vs. conservative PK/PD target on the clinical efficacy of beta-lactams for the treatment of Gram-negative infections among the critically ill patients; (b) identifying the patient’s conditions associated with failure in attaining aggressive PK/PD targets.

The meta-analysis was registered in the PROSPERO database (CRD42023494380) and conducted according to the Preferred Reporting Items for Systematic Review and Meta-Analyses (PRISMA) guideline [13].

### PICO 1 question

*Population*: critically ill patients with documented or suspected Gram-negative infections treated with beta-lactams

*Intervention*: attainment of aggressive beta-lactam PK/PD targets

*Comparator*: attainment of conservative beta-lactam PK/PD targets

*Outcome*: clinical efficacy (i.e., clinical and microbiological outcome)

### PECO 2 question

*Population:* critically ill patients with documented or suspected Gram-negative infections treated with beta-lactams

*Exposure:* risk factors associated with failure in attaining aggressive beta-lactam PK/PD targets

*Comparator:* no risk factor associated with failure in attaining aggressive beta-lactam PK/PD targets

*Outcome:* attainment of aggressive PK/PD targets for beta-lactams in critically ill patients having specific risk factors assessed

### Search strategy

PubMed-MEDLINE and Scopus database were independently searched by two authors (MG and PGC) from inception to 23 December 2023, by means of a specific search string: (“beta-lactam” OR “beta-lactams” OR “piperacillin” OR “ceftazidime” OR “cefepime” OR “meropenem” OR “imipenem”) AND (“predictor” OR “risk factor” OR “underexposure” OR “target attainment” OR “pharmacokinetic/pharmacodynamics” OR “PK/PD” OR “therapeutic drug monitoring” OR “drug monitoring” OR “therapeutic monitoring” OR “TDM”). No language limitation was established. Two authors (MG and PGC) independently assessed retrieved records for duplicate removal. Reference lists of included studies were also screened for identifying potential articles fulfilling inclusion criteria.

### Study selection

Selected studies included randomized controlled trials (RCTs) and/or observational studies assessing the impact of attaining aggressive vs. conservative PK/PD targets on clinical efficacy of beta-lactams in the treatment of critically ill patients with documented or suspected Gram-negative infections and/or the risk factors associated with failure in attaining aggressive PK/PD targets. The PK/PD target of beta-lactam was considered aggressive whenever the reported free beta-lactam trough (C_min_) or steady-state concentrations (C_ss_) to MIC ratio was > 4 (equivalent to 100%*f*T_> 4xMIC_), in agreement with both preclinical/clinical studies and international guidance [4, 14]. In studies assessing beta-lactam/beta lactamase inhibitor combinations (BL/BLIc), the definition of aggressive PK/PD target attainment was based on a joint PK/PD target attainment of both the BL and the BLI, in agreement with previous studies [15].

Exclusion criteria were the lack of quantitative data for the different selected outcomes, of comparator group, or of analysis providing adjustment for confounders. Case series, case reports, and conference abstracts were also excluded.

Clinical cure was selected as the primary outcome for dealing with PICO 1 question. Microbiological failure, resistance development, mortality rate, and survival rate were assessed as the secondary outcomes.

Studies assessing potential predictors independently associated with failure in attaining aggressive beta-lactam PK/PD target after adjustment for confounders were included for dealing with PECO 2 question. Risk factors were categorized into four categories, namely demographics/clinical characteristics of the patients, pathophysiological alterations, beta-lactam PK features, and beta-lactam PD features in terms of isolated pathogens and susceptibility, according to the principles of the so-called “antimicrobial therapy puzzle” [16].

Screening of titles and abstracts of retrieved records was independently performed by two authors (MG and PGC). Potential discrepancies were resolved by means of discussion between the two authors.

### Data extraction

Relevant data were independently extracted by two authors (MG and PGC) from each of the included studies. Specifically, the following information were retrieved: study author and year of publication, study characteristics (study design, country), funding sources, demographics and clinical features of patients, site of infections, Gram-negative clinical isolates, type of beta-lactam and administration mode, outcome data.

In the eventuality that unclear and/or missing data were found in the included studies, the corresponding authors would have been contacted for clarification.

### Risk of bias assessment

Risk of bias for included studies was independently investigated by two authors (MG and PGC) according to the Cochrane Risk of Bias Tool (RoB 2.0) [17] and the Risk Of Bias In Non-randomized Studies of Interventions (ROBINS-I) [18] for RCTs and observational studies, respectively. Any disagreement was discussed with a third reviewer (FP).

### Data synthesis

For PICO 1, the impact of attaining aggressive vs. conservative beta-lactam PK/PD target on the primary and secondary outcomes of beta-lactam efficacy in the treatment of critically ill patients having Gram-negative infections was meta-analyzed by pooling the adjusted odds ratios (aORs) deriving from propensity score, matched cohorts, or multivariate logistic regression analyses extrapolated from the included studies, after providing adjustment for confounders.

For PECO 2, the patient’s conditions potentially associated with failure in attaining aggressive PK/PD targets of beta-lactams were meta-analyzed by pooling the aORs of independent risk factors of failure in attaining aggressive PK/PD targets extrapolated from the included studies providing multivariate logistic regression analyses. Only those risk factors having aOR and 95% confidence interval (CI) reported in at least two studies were included.

Treatment effects were calculated as OR with 95% CI for dichotomous data, by using a random-effect model with inverse variance method. Statistical significance was assessed by using a Z-test, and *p* values < 0.05 were considered statistically significant.

A predictive risk score of failure in attaining aggressive beta-lactam PK/PD targets in critically ill patients was developed by assigning to each of the meta-analyzed risk factor showing statistical significance a point value corresponding to the natural log of the estimate rounded to the nearest integer, as previously reported [19]. Positive point values were considered as increasing the risk, whereas the negative ones were considered as being protective against the risk. The specific patient’s individual total score may be obtained by summing the single point score of each of the significant variable.

Statistical heterogeneity among studies was assessed by χ^2^ test (*p* < 0.10 indicated significant heterogeneity) and *I*^2^ (> 50% indicated substantial heterogeneity). Publication bias was assessed by visual inspection of the funnel plot and Egger’s test. Sensitivity analysis was conducted according to the risk of bias, by excluding studies at high or serious/critical risk of bias.

Statistical analysis was performed by means of MedCalc for Windows (MedCalc statistical software, version 19.6.1, Ostend, Belgium).

## Results

### Literature search

A total of 20,364 potential studies were retrieved, and 20,326 out of these were excluded after searching for duplicates and after initial screening of titles and abstracts. Overall, 38 full-text articles were considered potentially eligible, and 21 out of these met the final inclusion criteria [the remaining 17 were excluded because of lack of adjusted outcome data (ten studies); assessing only conservative beta-lactam PK/PD targets (six studies), or assessing only TDM-guided approach (one study)] (Additional file 2: Fig. 1).

**Fig. 1:**
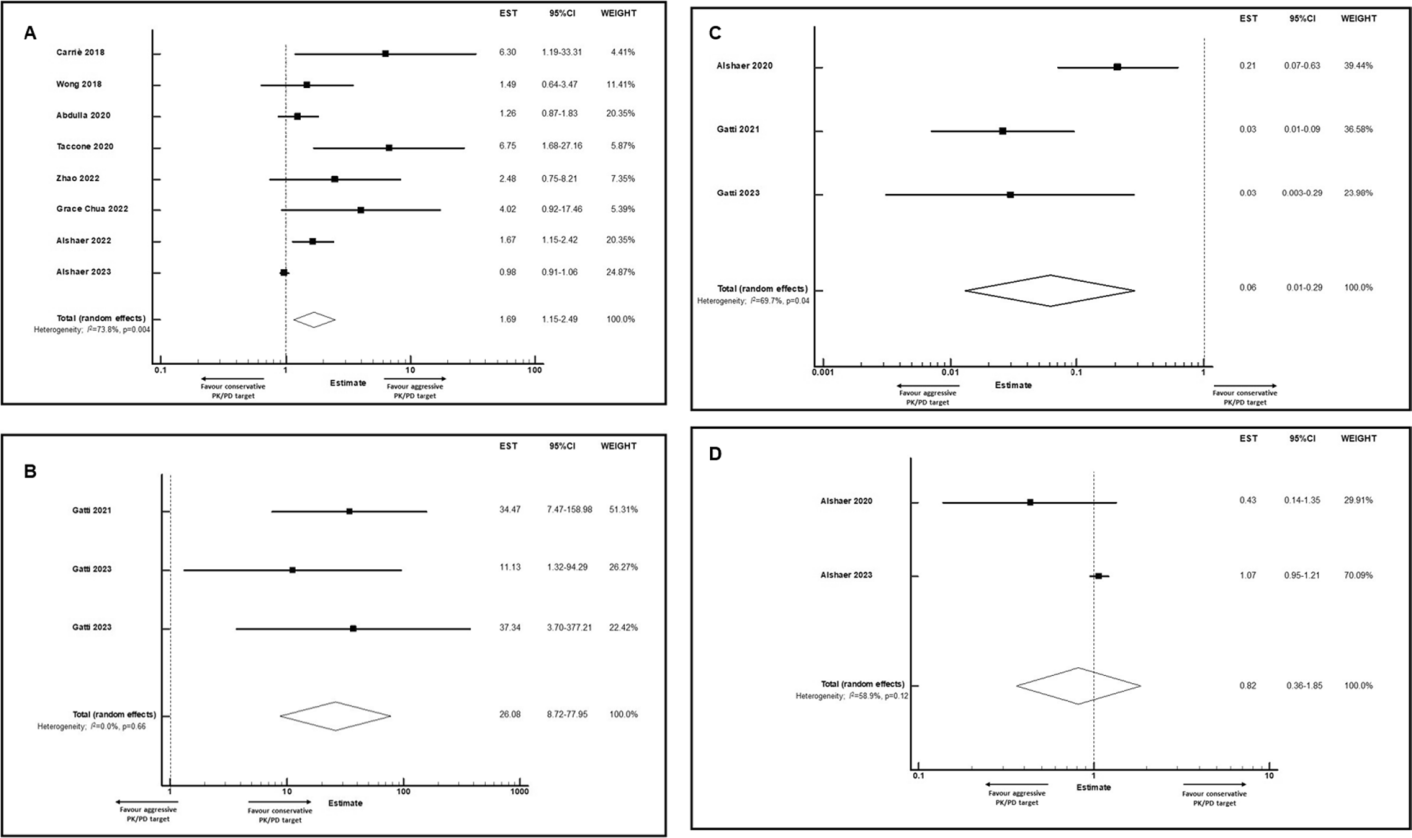
Forest plots of aOR showing clinical cure rate (**a**), microbiological failure rate (**b**), resistance occurrence (**c**), and mortality rate (**d**) in critically ill patients attaining aggressive vs. conservative PK/PD targets of beta-lactams

### Features of the included studies

The 21 observational studies included had a design that was prospective in 9 cases and retrospective in the other 12 (Table 1) [6, 7, 15, 20–37]. Five were multicentric [22, 25, 32, 35, 36]. Overall, a total of 4833 patients was enrolled (2193 attaining aggressive beta-lactam PK/PD targets vs. 2640 attaining conservative PK/PD targets). Since the patients extrapolated from two studies [21, 22] were meta-analyzed both in PICO 1 and PECO 2, the total number of included patients resulted of 2296 in PICO 1 and 2763 in PECO 2. Fourteen studies were conducted in Europe, three in the USA, and two each in Australia and Asia.

**Table 1:** Main features of the included studies

Study reference	Stud design	Country	Time period	No. of enrolled patients	Age (mean or median)^a^	Sex (Male)	Severity index	Beta-lactam and mode of administration	Type of infections	Isolated pathogens	Aggressive PK/PD taret (reference MIC value)	Timing of PK/PD target assessment
*PICO 1—Clinical efficacy of attaining aggressive vs. conservative beta-lactams PK/PD targets*												
Wong et al. [20]	Prospective observational monocentric	Australia	2012–2013	369	53.4 ± 17.7	66%	RRT 13.8% Median APACHE II 22 (16–27)	Ceftriaxone, cefazolin, meropenem, ampicillin, benzylpenicillin, flucloxacillin and piperacillin CI 4.3%	VAP 35.0% BSI 16.8% IAI 9.2%	NA	100*f*T_>4 x MIC_ (MIC value of the clinical isolate in case of targeted therapy; EUCAST clinical breakpoint in case of empirical therapy)	NS
Carriè et al.^b^ [21]	Prospective observational monocentric	France	2016–2017	79	52 (33–68)	78%	SAPS II 40 (27–47) Vasopressors 52% ARDS 19%	Cefazolin, cefotaxime, piperacillin/tazobactam, cefepime, ceftazidime, meropenem CI 100.0%	VAP 72% IAI 13% BSI 11% UTI 9%	*Enterobacteriaceae* 66% Non-fermenting GNB 11%	100*f*T_>4 x MIC_ (MIC value of the clinical isolate in case of targeted therapy; EUCAST clinical breakpoint in case of empirical therapy)	At 24 h, 48 h, and 72 h
Abdulla et al.^b^ [22]	Prospective observational multicentric	Netherlands	2016–2017	147	63 (56–70)	61.9%	Median SOFA 11 (7–15) Median APACHE II 23 (18–27) RRT 19%	Amoxicillin, cefotaxime, ceftazidime, ceftriaxone, cefuroxime, meropenem CI 0.0%	NA	NA	100*f*T_>4 x MIC_ (EUCAST clinical breakpoint)	At 48 h
Alshaer et al. [23]	Retrospective observational monocentric	USA	2016–2018	206	59 (46–69)	62%	Median SOFA 5 (2–8) Median APACHE II 17 (12–22) RRT 15%	Ampicillin, ceftriaxone, cefazolin, cefepime, meropenem, piperacillin CI 0.0%	VAP 57.3% BSI 31.1% UTI 12.6% SSTI 12.6% IAI 6.8% CNS 2.4%	*P. aeruginosa* 44.2% *K. pneumoniae* 19.9% *E. cloacae* 14.6% *E. coli* 14.1%	100*f*T_>4 x MIC_ (MIC value of the clinical isolate being all targeted therapies)	NS
Taccone et al. [24]	Retrospective observational monocentric	Belgium	2010–2016	70	55 (41–61)	49%	Lung transplant recipients 100.0% Median SOFA 9 (7–11) Median APACHE II 14 (12–19) RRT 13% ECMO 23% MV 59% Vasopressors 43%	Cefepime, piperacillin, meropenem CI 0.0%	VAP 42.9% UTI 2.9% IAI 1.4% CR-BSI 1.4%	*P. aeruginosa* 11.4% *E. cloacae* 7.1% *K. aerogenes* 7.1% *E. coli* 5.7% *S. maltophilia* 4.3%	100*f*T_>4 x MIC_ (EUCAST clinical breakpoint for *P. aeruginosa*)	NS
Gatti et al. [7]	Retrospective observational monocentric	Italy	2020–2021	116	66 (56–73)	69.8%	Vasopressors 53.5% MV 87.1% RRT 22.4%	Ceftazidime, meropenem, piperacillin/tazobactam CI 100.0%	VAP 49.1% BSI 28.1% UTI 11.2% IAI 1.7% BJI 0.9% CNS 0.9%	*K. pneumoniae* 25.7% *P. aeruginosa* 23.7% *E. coli* 19.4% *Enterobacter spp* 10.1% *P. mirabilis* 5.0% Other 16.6%	C_ss_/MIC > 5 (MIC value of the clinical isolate being all targeted therapies)	Within 72 h
Chua et al. [25]	Prospective observational multicentric	Singapore	2016–2018	85	61.3 ± 14.8	80.0%	Median APACHE II 25 (20–31) RRT 44% MV 82% Vasopressors 56% ECMO 18%	Meropenem, piperacillin/tazobactam CI 1% EI (over 3-4 h) 60.9%	VAP 69% BSI 21% IAI 8%	NA	100*f*T_>5 x MIC_ (MIC value of the clinical isolate in case of targeted therapy; EUCAST clinical breakpoint *for P. aeruginosa* in case of empirical therapy)	Within 48 h
Zhao et al. [26]	Prospective observational monocentric	China	2019	64	64 (51–71)	73.4%	Median APACHE II 17 (12–23)	Meropenem CI 0.0% (6.3% EI > 2 h)	NA	NA	C_min_/MIC > 4 Punctual MIC values (all targeted therapies)	Within 48 h
Alshaer et al. [27]	Retrospective observational monocentric	USA	2016–2021	840	56 ± 20	61%	Mean SOFA 6 ± 4 RRT 21%	Meropenem, cefepime, piperacillin/tazobactam CI 0.0%	HAP/VAP 100.0%	*P. aeruginosa* 44.3% *Enterobacter spp* 6.8% *K. pneumoniae* 6.2% *S. marcescens* 5.0% *E. coli* 4.9% *A. baumannii* 3.8%	100*f*T_>4 x MIC_ Punctual MIC values (all targeted therapies)	At 24 h, at day 10, and at the end of therapy
Gatti et al. [28]	Retrospective observational monocentric	Italy	2021–2023	58	62.5 (55.5–73.8)	62.1%	RRT 25.9% ARC 10.3%	Ceftazidime/avibactam CI 100.0%	BSI 41.4% VAP 19.0% VAP + BSI 17.2% IAI + BSI 12.1% IAI 5.2% Other 5.1%	KPC-Kp 31.0% OXA-48-producing *Enterobacterales* 27.6% DTR-PA 24.1%	*f*C_ss_/MIC > 4 (ceftazidime) *f*C_ss_/C_T_ > 1 (avibactam) (MIC value of the clinical isolate being all targeted therapies)	At 72 h for first assessment
Alshaer et al. [6]	Retrospective observational monocentric	USA	2016–2021	213	58 ± 16	57.0%	Mean SOFA 8 ± 4 RRT 24%	Cefepime, meropenem, piperacillin/tazobactam CI 0.0%	BSI 100.0% CI 11%	*P. aeruginosa* 36.2% *E. coli* 19.2% *K. pneumoniae* 16.0% Other 28.6%	100*f*T_>4 x MIC_ (MIC value of the clinical isolate being all targeted therapies)	At 24 h and at day 7
Gatti et al. [29]	Retrospective observational monocentric	Italy	2021–2023	43	69 (57–74)	58.1%	Median SOFA 8 (4–11) MV 81.4% Vasopressors 62.8% RRT 25.6% ARC 7.0%	Piperacillin/tazobactam CI 100.0%	BSI 55.8% VAP 37.2% VAP + BSI 7.0%	*E. coli* 37.5% *P. aeruginosa* 29.0% *K. pneumoniae* 12.5% Other 21.0%	*f*C_ss_/MIC > 4 (piperacillin) *f*C_ss_/C_T_ > 1 (tazobactam) (MIC value of the clinical isolate being all targeted therapies)	NS
*PECO 2—Predictive factors for failure in attaining aggressive beta-lactams PK/PD targets*												
Udy et al. [30]	Retrospective observational monocentric	Australia	NA	52	52.9 ± 20.9	70.8%	Mean APACHE II 20.3 ± 6.8 Mean SAPS II 39.6 ± 16.1 MV 85% Vasopressors 25%	Ampicillin, dicloxacillin, penicillin, flucloxacillin, piperacillin, cephalothin, cefazolin, ceftriaxone, ceftazidime, cefepime, meropenem, ertapenem CI 0.0%	VAP 52%	*Enterobacterales* 29.1% *Pseudomonas spp* 3.6% *Acinetobacter spp* 1.8% Empirical therapy 30.9%	100*f*T_>4 x MIC_ (MIC value of the clinical isolate in case of targeted therapy; EUCAST clinical breakpoint in case of empirical therapy)	NS
Hites et al. [31]	Retrospective observational monocentric	Belgium	2009–2011	68	59 (24–79)	55%	Median APACHE II 18 (8–32) Median SOFA 10 (1–19) RRT 50% MV 47% Vasopressors 72%	Cefepime, meropenem, piperacillin/tazobactam CI 0.0%	VAP 37% IAI 31% SSTI 27% UTI 6%	*P. aeruginosa* 29% *Enterobacterales* 29% Non-fermenting GNB 18%	100*f*T_>4 x MIC_ (EUCAST clinical breakpoint for *P. aeruginosa*)	NS
Alobaid et al. [32]	Retrospective observational multicentric	Australia, Germany, Spain	NA	1400	67 (52–76)	65%	BMI > 30 27.1%	Piperacillin/tazobactam, meropenem CI 54.6%	NA	NA	100*f*T_>4 x MIC_ (EUCAST clinical breakpoint for *P. aeruginosa*)	NS
Damen et al. [33]	Prospective observational monocentric	Belgium	2016–2018	356	66 (55–74)	66.6%	Median APACHE II 23.0 (20.8–26.0) Median SOFA 6.0 (2.5–9.0) ARC 41.9%	Piperacillin/tazobactam CI 100.0%	NA	NA	100*f*T_>4 x MIC_ (EUCAST clinical breakpoint for *P. aeruginosa*)	21.1% at 24 h, 35.1% at 48 h, 43.8% after > 48 h
Dhaese et al. [34]	Prospective observational monocentric	Belgium	NA	253	62.2 ± 15.0	64.8%	Mean APACHE II 23.5 ± 8.4 Median SOFA 5 (0–9) Vasopressors 1%	Piperacillin/tazobactam, meropenem CI 100.0%	NA	NA	100*f*T_>4 x MIC_ (EUCAST clinical breakpoint for *P. aeruginosa*)	At 48 h
Fillatre et al. [35]	Prospective observational multicentric	France	NA	42	60 (49–66)	81%	Median SAPS II 50 (40–65) Median SOFA 11 (9–14) ECMO 50%	Piperacillin/tazobactam EI 4 h 100.0%	NA	*Enterobacterales* 26.2% *P. aeruginosa* 11.9% Empirical therapy 40.5%	100*f*T_>4 x MIC_ (EUCAST clinical breakpoint for *P. aeruginosa*)	NS
Guilhaumou et al. [36]	Prospective observational multicentric	France	2015–2017	196	56 (53.6–58.4)	64.7%	Median SAPS II 39 (38.5–51) Median SOFA 4 (2–5) RRT 6%	Cefepime, cefotaxime, ceftazidime, meropenem CI 100.0%	VAP 51.5% BSI 25.7% UTI 15.7% CNS 2.2% IAI 0.7%	*E. coli* 26.1% *P. aeruginosa* 17.0% *K. pneumoniae* 13.7%	100*f*T_>4 x MIC_ (EUCAST clinical breakpoint)	At 24 h, at day 4, and at day 7
Gatti et al. [15]	Retrospective observational monocentric	*Italy*	*2021–2023*	*100*	*57 (51–63)*	63.6%	Orthotopic liver transplant recipients 100.0% Median SOFA 6.5 (3.75–9.25) MV 46.8% Vasopressors 64.9% RRT 36.4% ARC 19.5%	Meropenem, piperacillin/tazobactam, ceftazidime/avibactam, meropenem/vaborbactam CI 100.0%	VAP 39.5% IAI 25.6% BSI 20.9% IAI + BSI 9.3% VAP + BSI 4.7%	*K. pneumoniae* 31.3% *E. cloacae* 15.7% *E. coli* 13.7% *P. aeruginosa* 13.7%	*f*C_ss_/MIC > 4 (piperacillin; meropenem; ceftazidime) *f*C_ss_/C_T_ > 1 (tazobactam; avibactam) *f*AUC/C_T_ > 24 (vaborbactam) (MIC value of the clinical isolate in case of targeted therapy; EUCAST clinical breakpoint in case of empirical therapy)	NS
Tournayre et al. [37]	Retrospective observational monocentric	*France*	*2019–2020*	*70*	*69 (60–74)*	69%	Median SAPS II 54 (41–67) Median SOFA 8 (6–10) Septic shock 56% RRT 19% ARC 17%	Meropenem CI 52.9% EI 47.1%	VAP 53% UTI 19% IAI 13% BSI 10%	NA	C_min_ or C_ss_/MIC > 5 (EUCAST clinical breakpoint for *P. aeruginosa*)	At 48 h

*APACHE* acute physiologic assessment and chronic health evaluation, *ARC* augmented renal clearance, *ARDS* acute respiratory distress syndrome, *AUC* area under concentration-to-time curve, *BJI* bone and joint infection, *BMI* body mass index, *BSI* bloodstream infection, *CI* continuous infusion, *C*_*min*_ trough concentrations, *CNS* central nervous system, *CR-BSI* catheter-related bloodstream infections, *C*_*T*_ target concentrations, *DTR* difficult-to-treat resistant, *ECMO* extracorporeal membrane oxygenator, *EI* extended infusion, *fC*_*ss*_ free steady-state concentrations, *GNB* Gram-negative bacteria, *HAP* hospital-acquired pneumonia, *IAI* intrabdominal infection, *MIC* minimum inhibitory concentration, *MV* mechanical ventilation, *NA* not assessed, *NS* not specified, *PA* *Pseudomonas aeruginosa*, *RRT* renal replacement therapy, *SAPS* simplified acute physiology score, *SOFA* sequential organ failure assessment, *SSTI* skin and soft tissue infection, *UTI* urinary tract infection, *VAP* ventilator-associated pneumonia

^a^Data are presented as mean ± standard deviation or median (interquartile range), except for the study of Hites et al. [31] reporting median (range)

^b^Included also in PECO 2

Median and/or mean age ranged from 52 to 69 years, with a male preponderance (ranging from 55 to 81%) in all but one study. Meropenem (in 17/21 studies) and piperacillin/tazobactam (in 16/21 studies) were the two most frequently used beta-lactams. Beta-lactams were administered by continuous infusion (CI) in 11/21 studies, by prolonged infusion in 3/21 studies and by intermittent infusion in 7/21 studies. The beta-lactam dosing regimens adopted in the different studies are reported in the Additional file 1: Table 1. Hospital-acquired pneumonia (HAP) and/or ventilator-associated pneumonia (VAP) accounted for most of the infection types (13/21 studies).

The aggressive beta-lactam PK/PD target selected in the different studies was a 100%*f*T_>4x MIC_ and/or C_ss_ or C_min_/MIC ratio > 4 in 18/21 studies [6, 15, 20–24, 26–36], and a 100%*f*T_>5x MIC_ and/or C_ss_ or C_min_/MIC ratio > 5 in 3/21 studies [7, 25, 37]. Joint PK/PD target was assessed in three studies evaluating BL/BLIc (namely piperacillin-tazobactam, ceftazidime-avibactam, and meropenem-vaborbactam) [15, 28, 29]. In 11/21 studies, the assessment of aggressive PK/PD target attainment of beta-lactams was assessed first within 72 h from starting treatment.

### Impact of attaining aggressive vs. conservative PK/PD targets on the clinical efficacy of beta-lactams

A summary of outcome definition for each included study is reported in Table 2.

**Table 2:** Summary of outcome definition for each included study in PICO 1

Study	Clinical cure	Microbiological failure	Beta-lactam resistance occurrence	Mortality rate	Survival rate
Wong et al. [20]	Resolution (disappearance of all signs and symptoms related to the infection) or improvement (marked or moderate reduction in the severity and/or number of signs and symptoms of infection) clinically as documented by independent clinicians in patients’ progress notes	Not assessed	Not assessed	Not assessed	Not assessed
Carriè et al. [21]	Favourable clinical response (resolution of fever, organ dysfunction, clinical and biological symptoms of the initial infection) with no need for escalating antibiotics during treatment and/or within 15 days after end of treatment. Superinfections due to new causative pathogens with natural resistance to the initial antimicrobial therapy were not considered as therapeutic failure	Not assessed	Not assessed	Not assessed	Not assessed
Abdulla et al. [22]	Reduction in ICU length of stay	Not assessed	Not assessed	Not assessed	Survival rate at 30-day after starting antibiotic therapy
Alshaer et al. [23]	Not assessed	Not assessed	Development of resistance of a Gram-negative organism to the original selected beta-lactam, to which it was susceptible	In-hospital mortality rate	Not assessed
Taccone et al. [24]	Lack of acquisition of early Gram-negative infections and/or early MDR acquisition or infection (i.e., within 14 days after the transplantation)	Not assessed	Not assessed	Not assessed	Not assessed
Gatti et al. [7]	Not assessed	Persistence of the same gram-negative pathogen isolated from index culture after ≥ 7 days from starting beta-lactam treatment	The increase of the MIC of the clinical isolate beyond the EUCAST clinical breakpoint for the specific selected beta-lactam	Not assessed	Not assessed
Chua et al. [25]	Improvement in presenting signs and symptoms of infection and/or inflammatory markers, and/or discontinuation, de-escalation, or oral conversion of initial beta-lactam therapy	Not assessed	Not assessed	Not assessed	Survival rate at 14-day
Zhao et al. [26]	Disappearance of all signs and symptoms related to infection or a marked or moderate reduction in the severity and/or number of signs and symptoms of infection	Not assessed	Not assessed	Not assessed	Not assessed
Alshaer et al. [27]	Resolution of infection-related symptoms present at the start of therapy, the resolution or lack of progression of radiological signs of pneumonia without change or addition of antibiotic therapy, and non-initiation of a new antibiotic within 48 h of stopping the original one	Not assessed	Not assessed	Not assessed	Survival rate at 28-day
Gatti et al. [28]	Not assessed	Persistence of the same gram-negative pathogen isolated from index culture after ≥ 7 days from starting beta-lactam treatment	Not assessed	Not assessed	Not assessed
Alshaer et al. [6]	Resolution of infection-related symptoms at day-7	Not assessed	Not assessed	30-day mortality rate	Not assessed
Gatti et al. [29]	Not assessed	Persistence of the same gram-negative pathogen isolated from index culture after ≥ 7 days from starting beta-lactam treatment	The increase of the MIC of the clinical isolate beyond the EUCAST clinical breakpoint for the specific selected beta-lactam	Not assessed	Not assessed

*ICU* intensive care unit, *MDR* multidrug-resistant

Results of meta-analysis for the primary and the secondary outcomes are summarized in Table 3.

**Table 3:** Results of meta-analysis for primary and secondary outcomes of attaining aggressive vs. conservative PK/PD targets of beta-lactams in critically ill patients

Outcome	Studies	PK/PD target assessed	No. of patients (Aggressive vs. conservative PK/PD targets)	Odds ratio (95% CI)	Heterogeneity (*I*^2^; *p* value)	Publication bias (*p* value Egger’s test)
Clinical cure	8	100%*f*T_>4–5 x MIC_ *f*C_ss_/MIC > 4–5	701 vs. 1172	1.69 (1.15–2.49) *p* = 0.007	73.8% *p* = 0.004	0.10
Microbiological failure	3	*f*C_ss_/MIC < 4–5	175 vs. 42	26.08 (8.72–77.95) *p* < 0.001	0.0% *p* = 0.66	0.73
Resistance occurrence	3	100%*f*T_>4–5 x MIC_ *f*C_ss_/MIC > 4–5	269 vs. 96	0.06 (0.01–0.29) *p* < 0.001	69.7% *p* = 0.04	0.62
Mortality rate	2	100%*f*T_>4 x MIC_	269 vs. 150	0.82 (0.36–1.85) *p* = 0.63	58.9% *p* = 0.12	NA
Survival rate	3	100%*f*T_>4–5 x MIC_	313 vs. 759	1.15 (0.50–2.66) *p* = 0.75	66.2% *p* = 0.05	0.33

*CI* confidence interval, *C*_*ss*_ steady-state concentration, *MIC* minimum inhibitory concentration, *NA* not applicable, *PK/PD* pharmacokinetic/pharmacodynamic

Eight studies (1873 patients) provided data for assessing clinical cure in critically ill patients treated with beta-lactams [6, 20–22, 24–27]. Overall, attaining aggressive PK/PD targets was significantly associated with higher clinical cure rate (OR 1.69; 95% CI 1.15–2.49; *p* = 0.007; Fig. 1a). The degree of heterogeneity was substantial (*I*^2^ = 73.8%; *p* = 0.004), and no evidence of publication bias was found (*p* = 0.10).

Three studies (217 patients) provided data for assessing microbiological outcome [7, 28, 29]. Overall, failure in attaining aggressive beta-lactam PK/PD targets was significantly associated with higher risk of microbiological failure (OR 26.08; 95% CI 8.72–77.95; *p* < 0.001; Fig. 1b). Substantial heterogeneity (*I*^2^ = 0.0%) and publication bias (*p* = 0.73) were not reported.

Three studies (365 patients) provided data for assessing beta-lactam resistance occurrence [7, 23, 29]. Overall, attaining aggressive PK/PD targets was significantly associated with lower risk of beta-lactam resistance development (OR 0.06; 95%CI 0.01–0.29; *p* < 0.001; Fig. 1c). A substantial degree of heterogeneity was observed (*I*^2^ = 69.7%; *p* = 0.04), and no evidence of publication bias was found (*p* = 0.62).

Mortality and survival rate were assessable based on two [6, 23] and three studies [22, 25, 27], respectively, accounting for a total of 419 and 1,072 included critically ill patients, respectively. Overall, attaining aggressive PK/PD targets was not significantly associated neither with lower risk of mortality rate (OR 0.82; 95% CI 0.36–1.85; *p* = 0.63; Fig. 1d), nor with higher survival rate (OR 1.15; 95% CI 0.50–2.66; *p* = 0.75; Additional file 3: Fig. 2).

### Predictors of failure in attaining aggressive PK/PD targets of beta-lactams

Nine risk factors belonging to the four predefined categories met the inclusion criteria of being investigated in at least two studies and were meta-analyzed as potential predictors (Table 4).

**Table 4:** Predictive factors of failure in attaining aggressive beta-lactam PK/PD targets in critically ill patients

Risk factor	Studies	No. of patients (aggressive vs. conservative PK/PD targets)	Odds ratio (95% CI)	Heterogeneity (*I*^2^; *p* value)	Publication bias (*p* value Egger’s test)	Log estimate	Point score
Age	4	598 vs. 1001	1.00 (0.98–1.02) *p* = 0.95	49.8% *p* = 0.11	0.63	NA	0
Male gender	3	582 vs. 965	0.34 (0.25–0.48) *p* < 0.001	0.0% *p* = 0.38	0.06	-1.08	1
BMI > 30 kg/m^2^	3	582 vs. 965	0.92 (0.85–0.99) *p* = 0.032	0.0% *p* = 0.76	0.18	-0.08	1
eGFR	4	224 vs. 298	0.98 (0.95–1.00) *p* = 0.07	79.8% *p* = 0.002	0.05	NA	0
Prolonged infusion	2	331 vs. 220	7.54 (4.49–12.68) *p* < 0.001	0.0% *p* = 0.56	NA	2.02	− 2
Daily dose	3	590 vs. 880	1.09 (0.92–1.30) *p* = 0.32	54.6% *p* = 0.11	0.50	NA	0
Augmented renal clearance	2	151 vs. 28	9.02 (2.97–27.39) *p* < 0.001	0.0% *p* = 0.81	NA	2.20	2
SOFA	2	193 vs. 205	0.82 (0.43–1.59) *p* = 0.56	34.7% *p* = 0.22	NA	NA	0
MIC value above the clinical breakpoint	2	151 vs. 28	18.47 (1.22–278.86) *p* = 0.035	71.5% *p* = 0.06	NA	2.92	2

*BMI* body mass index, *CI* confidence interval, *eGFR* estimated glomerular filtration rate, *MIC* minimum inhibitory concentration, *NA* not applicable, *PK/PD* pharmacokinetic/pharmacodynamics, *SOFA* sequential organ failure assessment

**Fig. 2:**
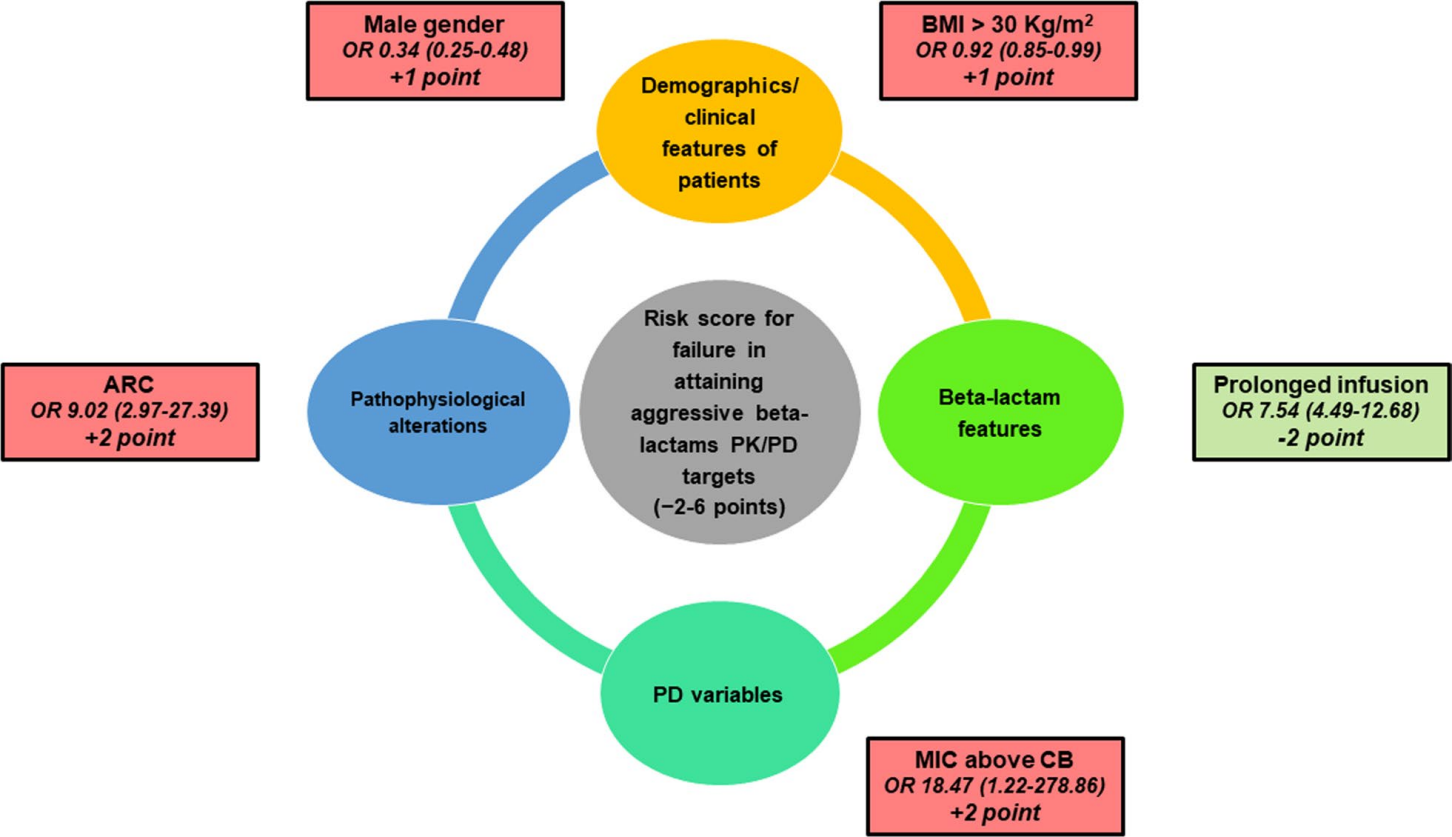
Significant independent predictors of failure in attaining aggressive PK/PD targets of beta-lactams. A risk score ranging from − 2 to 6 points was developed and proposed. ARC: augmented renal clearance; BMI: body mass index; CB: clinical breakpoint; MIC: minimum inhibitory concentration; OR: odds ratio; PK/PD: pharmacokinetic/pharmacodynamic

Five out of these resulted significantly associated with attaining aggressive PK/PD targets of beta-lactams, four by increasing the risk, and one as being protective against the risk. Specifically, male gender (N = 3; OR 0.34; 95% CI 0.25–0.48; *I*^2^ = 0.0%; Additional file 4: Fig. 3), body mass index (BMI) > 30 kg/m^2^ (N = 3; OR 0.92; 95% CI 0.85–0.99; *I*^2^ = 0.0%; Additional file 5: Fig. 4), augmented renal clearance (ARC) (N = 2; OR 9.02; 95% CI 2.97–27.39; *I*^2^ = 0.0%; Additional file 6: Fig. 5), and MIC values above the clinical breakpoint (N = 2; OR 18.47; 95% CI 1.22–278.86; *I*^2^ = 71.5%; Additional file 7: Fig. 6) emerged as significant independent predictors of failure in attaining aggressive beta-lactams PK/PD targets. Conversely prolonged/continuous infusion administration of beta-lactams resulted significantly protective against this risk (N = 2; OR 7.54; 95% CI 4.49–12.68; *I*^2^ = 0.0%; Additional file 8: Fig. 7). No significant publication bias emerged for any of the investigated predictors. Point assignment to these five independent predictors based on the natural log of the estimate resulted in a predictive risk score ranging from − 2 to 6 (Fig. 2).

### Sensitivity analysis

After excluding studies at serious/critical risk of bias, aggressive beta-lactams PK/PD target attainment resulted still associated with higher clinical cure rate (N = 6; OR 1.64; 95% CI 1.21–2.24), and the heterogeneity consistently decreased in comparison with the primary analysis (*I*^2^ = 20.2%; *p* = 0.28). No significant difference in secondary outcomes for PICO 1 emerged in comparison with the primary analysis.

Since none of the studies included in the primary analysis for PECO 2 showed a serious/critical risk of bias, no difference emerged in the sensitivity analysis.

### Quality of the included studies

The risk of bias in at least one domain was serious in 2/21 studies (bias in measurement of outcome was mostly reported), and moderate 19/21 (Additional file 1: Table 2).

## Discussion

To the best of our knowledge, this is the first study that meta-analyzed the clinical impact of attaining aggressive vs. conservative PK/PD target on the clinical efficacy of beta-lactams for the treatment of Gram-negative infections in the critically ill patients. Notably, attainment of aggressive PK/PD target of beta-lactams was significantly associated with both better clinical cure rate and lower risk of resistance development, whereas non-attainment significantly increased the risk of microbiological failure, although no clinical impact on mortality and/or survival rate emerged.

Indeed, the topic of which PK/PD target threshold could be the best for maximizing beta-lactam efficacy in the treatment of Gram-negative infections in the critically ill patients is a matter of ongoing debate [9]. Importantly, previous preclinical studies showed that PK/PD target attainment of C_min_/MIC ratios ranging between 3.8 and 6.2 with beta-lactams was effective in preventing the emergenge of breakthrough resistance to beta-lactams among Gram-negatives [38]. This represented a first rationale for starting the adoption of an aggressive PK/PD target of 100%*f*T_>4x MIC_ in clinical practice, as recently reported by several studies [6, 28, 29]. Scheduled timing for assessing aggressive PK/PD target attainment of beta-lactams may be crucial, especially in case of severe infections. Some guidance recommended that aggressive PK/PD target attainment should be assessed promptly when dealing with patients having sepsis and/or septic shock [14, 39]. In this regard, it should be noticed that almost two-thirds of the studies included in PICO 1 fully fulfilled with this recommendation by assessing first aggressive PK/PD target attainment of beta-lactams within 72 h from starting treatment.

It should also be recognized that the need of attaining aggressive PK/PD target of beta-lactams may be affected by some underlying conditions, namely the infection site and/or the magnitude of the bacterial load. This approach should be recommended especially when dealing with deep-seated infections having high-bacterial load, namely HAP and/or VAP, and could be less needed in case of urinary tract infections having low bacterial load [40, 41]. Our meta-analysis first, by providing strong evidence that aggressive PK/PD target attainment may increase clinical efficacy of beta-lactams in terms of both clinical and microbiological outcome, may support the definitive adoption of this aggressive PK/PD target in routine clinical practice when treating Gram-negative infections among the critically ill patients, as recently proposed by some guidance [4, 8, 14].

As a consequence of this, non-attaining aggressive PK/PD target of beta-lactams, by being resulted significantly associated with an increased risk of microbiological failure, should be prevented as much as possible. Previous studies included in a recent narrative review showed that several factors may favor non-attaining of both conservative and aggressive PK/PD targets of beta-lactams [12]. Our meta-analysis is in agreement with most of these [12], as it showed that male gender, morbid obesity (namely BMI > 30 kg/m^2^), ARC and in vitro resistance of the bacterial pathogen (namely MIC values above the clinical breakpoint) emerged as significant independent predictors of non-attaining aggressive PK/PD targets of beta-lactams. Conversely, prolonged/continuous infusion administration of beta-lactams resulted significantly protective against this risk. The added-value of our meta-analysis in this regard is that we first proposed a predictive risk score as helpful tool for supporting clinicians in promptly identifying which critically ill patients receiving standard beta-lactams dosing regimens could be at high-risk of non-attaining aggressive PK/PD target. The patient profile at the highest risk resulted that of a morbidly obese critically ill male with ARC having a Gram-negative related infection caused by an in vitro non-susceptible pathogen treated with a beta-lactam administered by intermittent infusion. In this scenario, implementing a real-time TDM-guided dosing adaptative strategy may provide valuable support in increasing the likelihood of early attaining and subsequently maintaining over time the aggressive PK/PD target, as recently shown [11].

Limitations of our meta-analysis should be recognized. No RCT was found in the literature search to be included neither for PICO 1 nor for PECO 2. The meta-analysis was based on observational studies, often with a retrospective design. This contributed to a moderate risk of bias in most cases, so that the findings should be interpreted cautiously. The choice of including only predictive factors being assessed in at least two studies could not rule out the possibility that some other relevant predictors of failure in attaining aggressive beta-lactams PK/PD targets might have been inadvertently excluded from our score. No subgroup analysis based on clinical feature differences (e.g., type of beta-lactam, infection site, specific pathogens) was feasible due to unavailability of data. The impact of an effective source control on clinical outcome could not be ruled out due to lacking data. The reliability of the predictive risk score should be necessarily prospectively validated in a large cohort of critically ill patients. Assessing risk of bias in the results of observational studies that compared the effects of different PK/PD targets by means of the ROBINS-I tool could have been less accurate than using dedicated tools for this purpose. Conversely, including only studies providing adjusted outcome data and investigating independent predictive factors of failure in attaining aggressive beta-lactams PK/PD targets represent a strength of our analysis, possibly minimizing the risk of confounding bias.

In conclusion, our meta-analysis showed that, after applying appropriate adjustments for confounders, aggressive PK/PD target attainment was significantly associated with higher clinical cure rate, lower microbiological failure rate, and lower risk of resistance development in critically ill patients receiving beta-lactams for documented or suspected Gram-negative infections. The developed predictive risk score of failure in attaining aggressive beta-lactams PK/PD targets should hopefully help clinicians in identifying patients at high-risk. Further analyses are warranted for confirming the findings and validating the proposed risk score.

### Supplementary Information

**Additional file 1. Supplementary Table 1.** Beta-lactam dosing regimens adopted in the included studies. **Supplementary Table 2**. Risk of bias assessment for observational studies according to ROBINS-I tool.

**Additional file 2. Supplementary Figure 1. **PRISMA flow diagram for study selection.

**Additional file 3. Supplementary Figure 2. **Forest plot of survival rate in critically ill patients attaining aggressive vs. conservative beta-lactams PK/PD targets.

**Additional file 4. Supplementary Figure 3. **Forest plot of the predictive factor male gender for failure in attaining aggressive beta-lactams PK/PD targets.

**Additional file 5. Supplementary Figure 4. **Forest plot of the predictive factor BMI > 30 Kg/m^2^ for failure in attaining aggressive beta-lactams PK/PD targets.

**Additional file 6. Supplementary Figure 5. **Forest plot of the predictive factor ARC for failure in attaining aggressive beta-lactams PK/PD targets.

**Additional file 7. Supplementary Figure 6. **Forest plot of the predictive factor MIC above clinical breakpoint for failure in attaining aggressive beta-lactams PK/PD targets.

**Additional file 8. Supplementary Figure 7.** Forest plot of the predictive factor prolonged infusion for attaining aggressive beta-lactams PK/PD targets.

## Data Availability

All data and materials generated during the current study are available from the corresponding author on reasonable request.

## References

[1] Angus DC, van der Poll T (2013). Severe sepsis and septic shock. N Engl J Med.

[2] Vincent J-L, Sakr Y, Singer M, Martin-Loeches I, Machado FR, Marshall JC (2020). Prevalence and outcomes of infection among patients in intensive care units in 2017. JAMA.

[3] Evans L, Rhodes A, Alhazzani W, Antonelli M, Coopersmith CM, French C (2021). Surviving sepsis campaign: international guidelines for management of sepsis and septic shock 2021. Intensive Care Med.

[4] Abdul-Aziz MH, Alffenaar J-WC, Bassetti M, Bracht H, Dimopoulos G, Marriott D (2020). Antimicrobial therapeutic drug monitoring in critically ill adult patients: a position paper. Intensive Care Med.

[5] Tam VH, Chang K-T, Zhou J, Ledesma KR, Phe K, Gao S (2017). Determining β-lactam exposure threshold to suppress resistance development in Gram-negative bacteria. J Antimicrob Chemother.

[6] Alshaer MH, Maranchick N, Alexander KM, Manigaba K, Shoulders BR, Felton TW (2023). Beta-lactam target attainment and associated outcomes in patients with bloodstream infections. Int J Antimicrob Agents.

[7] Gatti M, Cojutti PG, Pascale R, Tonetti T, Laici C, Dell’Olio A (2021). Assessment of a PK/PD target of continuous infusion beta-lactams useful for preventing microbiological failure and/or resistance development in critically ill patients affected by documented gram-negative infections. Antibiotics.

[8] Gatti M, Pea F (2023). Jumping into the future: overcoming pharmacokinetic/pharmacodynamic hurdles to optimize the treatment of severe difficult to treat-Gram-negative infections with novel beta-lactams. Expert Rev Anti Infect Ther.

[9] Berry AV, Kuti JL (2022). Pharmacodynamic thresholds for beta-lactam antibiotics: a story of mouse versus man. Front Pharmacol.

[10] Vardakas KZ, Voulgaris GL, Maliaros A, Samonis G, Falagas ME (2018). Prolonged versus short-term intravenous infusion of antipseudomonal β-lactams for patients with sepsis: a systematic review and meta-analysis of randomised trials. Lancet Infect Dis.

[11] Pai Mangalore R, Ashok A, Lee SJ, Romero L, Peel TN, Udy AA (2022). Beta-lactam antibiotic therapeutic drug monitoring in critically ill patients: a systematic review and meta-analysis. Clin Infect Dis.

[12] Abdulla A, Ewoldt TMJ, Purmer IM, Muller AE, Gommers D, Endeman H (2021). A narrative review of predictors for β-lactam antibiotic exposure during empirical treatment in critically ill patients. Expert Opin Drug Metab Toxicol.

[13] Page MJ, McKenzie JE, Bossuyt PM, Boutron I, Hoffmann TC, Mulrow CD (2021). The PRISMA 2020 statement: an updated guideline for reporting systematic reviews. BMJ.

[14] Guilhaumou R, Benaboud S, Bennis Y, Dahyot-Fizelier C, Dailly E, Gandia P (2019). Optimization of the treatment with beta-lactam antibiotics in critically ill patients-guidelines from the French Society of Pharmacology and Therapeutics (Société Française de Pharmacologie et Thérapeutique-SFPT) and the French Society of Anaesthesia and Intensive Care Medicine (Société Française d’Anesthésie et Réanimation-SFAR). Crit Care.

[15] Gatti M, Rinaldi M, Laici C, Siniscalchi A, Viale P, Pea F (2023). Role of a real-time TDM-based expert clinical pharmacological advice program in optimizing the early pharmacokinetic/pharmacodynamic target attainment of continuous infusion beta-lactams among orthotopic liver transplant recipients with documented or suspected gram-negative infections. Antibiotics (Basel).

[16] Pea F, Viale P (2006). The antimicrobial therapy puzzle: could pharmacokinetic-pharmacodynamic relationships be helpful in addressing the issue of appropriate pneumonia treatment in critically ill patients?. Clin Infect Dis.

[17] Sterne JAC, Savović J, Page MJ, Elbers RG, Blencowe NS, Boutron I (2019). RoB 2: a revised tool for assessing risk of bias in randomised trials. BMJ.

[18] Sterne JA, Hernán MA, Reeves BC, Savović J, Berkman ND, Viswanathan M (2016). ROBINS-I: a tool for assessing risk of bias in non-randomised studies of interventions. BMJ.

[19] Gatti M, Bonazzetti C, Tazza B, Pascale R, Miani B, Malosso M (2023). Impact on clinical outcome of follow-up blood cultures and risk factors for persistent bacteraemia in patients with gram-negative bloodstream infections: a systematic review with meta-analysis. Clin Microbiol Infect.

[20] Wong G, Briscoe S, McWhinney B, Ally M, Ungerer J, Lipman J (2018). Therapeutic drug monitoring of β-lactam antibiotics in the critically ill: direct measurement of unbound drug concentrations to achieve appropriate drug exposures. J Antimicrob Chemother.

[21] Carrié C, Petit L, d’Houdain N, Sauvage N, Cottenceau V, Lafitte M (2018). Association between augmented renal clearance, antibiotic exposure and clinical outcome in critically ill septic patients receiving high doses of β-lactams administered by continuous infusion: a prospective observational study. Int J Antimicrob Agents.

[22] Abdulla A, Dijkstra A, Hunfeld NGM, Endeman H, Bahmany S, Ewoldt TMJ (2020). Failure of target attainment of beta-lactam antibiotics in critically ill patients and associated risk factors: a two-center prospective study (EXPAT). Crit Care.

[23] Al-Shaer MH, Rubido E, Cherabuddi K, Venugopalan V, Klinker K, Peloquin C (2020). Early therapeutic monitoring of β-lactams and associated therapy outcomes in critically ill patients. J Antimicrob Chemother.

[24] Taccone FS, Bogossian EG, Tironi RM, Antonucci E, Hites M, Knoop C (2021). Early β-lactam concentrations and infectious complications after lung transplantation. Am J Transpl.

[25] Chua NG, Loo L, Hee DKH, Lim TP, Ng TM, Hoo GSR (2022). Therapeutic drug monitoring of meropenem and piperacillin-tazobactam in the Singapore critically ill population—A prospective, multi-center, observational study (BLAST 1). J Crit Care.

[26] Zhao Y, Xiao C, Hou J, Wu J, Xiao Y, Zhang B (2022). C/MIC > 4: a potential instrument to predict the efficacy of meropenem. Antibiotics (Basel).

[27] Alshaer MH, Maranchick N, Bai C, Maguigan KL, Shoulders B, Felton TW (2022). Using machine learning to define the impact of beta-lactam early and cumulative target attainment on outcomes in intensive care unit patients with hospital-acquired and ventilator-associated pneumonia. Antimicrob Agents Chemother.

[28] Gatti M, Rinaldi M, Bonazzetti C, Gaibani P, Giannella M, Viale P (2023). Could an optimized joint pharmacokinetic/pharmacodynamic target attainment of continuous infusion ceftazidime-avibactam be a way to avoid the need for combo therapy in the targeted treatment of deep-seated DTR Gram-negative infections?. Antimicrob Agents Chemother.

[29] Gatti M, Rinaldi M, Tonetti T, Siniscalchi A, Viale P, Pea F (2023). Could an optimized joint pharmacokinetic/pharmacodynamic target attainment of continuous infusion piperacillin-tazobactam be a valuable innovative approach for maximizing the effectiveness of monotherapy even in the treatment of critically ill patients with documented extended-spectrum beta-lactamase-producing enterobacterales bloodstream infections and/or ventilator-associated pneumonia?. Antibiotics (Basel).

[30] Udy AA, Varghese JM, Altukroni M, Briscoe S, McWhinney BC, Ungerer JP (2012). Subtherapeutic initial β-lactam concentrations in select critically ill patients: association between augmented renal clearance and low trough drug concentrations. Chest.

[31] Hites M, Taccone FS, Wolff F, Cotton F, Beumier M, De Backer D (2013). Case-control study of drug monitoring of β-lactams in obese critically ill patients. Antimicrob Agents Chemother.

[32] Alobaid AS, Brinkmann A, Frey OR, Roehr AC, Luque S, Grau S (2016). What is the effect of obesity on piperacillin and meropenem trough concentrations in critically ill patients?. J Antimicrob Chemother.

[33] Damen C, Dhaese S, Verstraete AG, Stove V, De Waele JJ (2019). Subtherapeutic piperacillin concentrations in neurocritical patients. J Crit Care.

[34] Dhaese SAM, Thooft ADJ, Farkas A, Lipman J, Verstraete AG, Stove V (2019). Early target attainment of continuous infusion piperacillin/tazobactam and meropenem in critically ill patients: a prospective observational study. J Crit Care.

[35] Fillâtre P, Lemaitre F, Nesseler N, Schmidt M, Besset S, Launey Y (2021). Impact of extracorporeal membrane oxygenation (ECMO) support on piperacillin exposure in septic patients: a case-control study. J Antimicrob Chemother.

[36] Guilhaumou R, Chevrier C, Setti JL, Jouve E, Marsot A, Julian N (2023). β-Lactam pharmacokinetic/pharmacodynamic target attainment in intensive care unit patients: a prospective, observational, cohort study. Antibiotics (Basel).

[37] Tournayre S, Mathieu O, Villiet M, Besnard N, Brunot V, Daubin D (2023). Factors associated with meropenem pharmacokinetic/pharmacodynamic target attainment in septic critically ill patients treated with extended intermittent infusion or continuous infusion. Int J Antimicrob Agents.

[38] Sumi CD, Heffernan AJ, Lipman J, Roberts JA, Sime FB (2019). What antibiotic exposures are required to suppress the emergence of resistance for gram-negative bacteria? A systematic review. Clin Pharmacokinet.

[39] Gatti M, Pea F (2023). The expert clinical pharmacological advice program for tailoring on real-time antimicrobial therapies with emerging TDM candidates in special populations: how the ugly duckling turned into a swan. Expert Rev Clin Pharmacol.

[40] Felton TW, Goodwin J, O’Connor L, Sharp A, Gregson L, Livermore J (2013). Impact of Bolus dosing versus continuous infusion of Piperacillin and Tazobactam on the development of antimicrobial resistance in Pseudomonas aeruginosa. Antimicrob Agents Chemother.

[41] Gatti M, Cojutti PG, Bartoletti M, Tonetti T, Bianchini A, Ramirez S (2022). Expert clinical pharmacological advice may make an antimicrobial TDM program for emerging candidates more clinically useful in tailoring therapy of critically ill patients. Crit Care.

