# Supplementary material for: Impact of attaining aggressive vs. conservative PK/PD target on the clinical efficacy of beta-lactams for the treatment of Gram-negative infections in the critically ill patients: a systematic review and meta-analysis
Source: Crit Care. 2024 Apr 16;28:123. doi: 10.1186/s13054-024-04911-5 (PMC11020314; doi:10.1186/s13054-024-04911-5)
Supplement: Supplementary file 1 — Additional file 1. Supplementary Table 1. Beta-lactam dosing regimens adopted in the included studies. Supplementary Table 2. Risk of bias assessment for observational studies according to ROBINS-I tool. [file 13054_2024_4911_MOESM1_ESM.docx]

**Supplementary materials**

**Supplementary Table 1 –** Beta-lactam dosing regimens adopted in the included studies.

**Supplementary Table 2 –** Risk of bias assessment for observational studies according to ROBINS-I tool.

**Supplementary Figure 1 –** PRISMA flow diagram for study selection.

**Supplementary Figure 2 –** Forest plot of survival rate in critically ill patients attaining aggressive vs. conservative beta-lactams PK/PD targets.

**Supplementary Figure 3 –** Forest plot of the predictive factor male gender for failure in attaining aggressive beta-lactams PK/PD targets.

**Supplementary Figure 4 –** Forest plot of the predictive factor BMI > 30 Kg/m^2^ for failure in attaining aggressive beta-lactams PK/PD targets.

**Supplementary Figure 5 –** Forest plot of the predictive factor ARC for failure in attaining aggressive beta-lactams PK/PD targets.

**Supplementary Figure 6 –** Forest plot of the predictive factor MIC above clinical breakpoint for failure in attaining aggressive beta-lactams PK/PD targets.

**Supplementary Figure 7 –** Forest plot of the predictive factor prolonged infusion for attaining aggressive beta-lactams PK/PD targets.

**Supplementary Table 1 –** Beta-lactam dosing regimens adopted in the included studies

| **Study** | **Included beta-lactams** | **Dosing regimens** | **TDM-guided dosing adjustments** |
| --- | --- | --- | --- |
| Wong *et al*., 2018 | Ceftriaxone  Cefazolin  Meropenem  Ampicillin  Benzylpenicillin Flucloxacillin  Piperacillin | 1g q12h – 2g q8h  1g q6h – 2g q8h  0.5g q12h – 2g q6h  1g q12h – 2g q4h  1.2g q4h – 2.4g q4h  2g q12h – 2g q2h  4.5g q12h – 4.5g q4h | Yes |
| Carriè *et al*., 2018 | Cefazolin  Cefotaxime  Piperacillin/tazobactam  Cefepime  Ceftazidime  Meropenem | 100 mg/kg/day  6g  18g  6g  6g  2g q8h | No |
| Abdulla *et al*., 2020 | Amoxicillin  Cefotaxime  Ceftazidime  Ceftriaxone  Cefuroxime  Meropenem | 6g (4g – 6g)  4g (4g – 4g)  3g (2.5g – 5g)  2g (2g – 2g)  4.5g (4.5g – 4.5g)  3g (2g – 3g) | No |
| Alshaer *et al*., 2020 | Ampicillin  Ceftriaxone  Cefazolin  Cefepime  Meropenem  Piperacillin | 4g  2g  6g (2g – 6g)  6g (1g – 6g)  4g (1g – 12g)  13.5g (6.75g – 18g) | No |
| Taccone *et al*., 2021 | Cefepime  Piperacillin  Meropenem | 2g q8h  4.5g q6h  1g q8h | No |
| Gatti *et al*., 2021 | Ceftazidime  Meropenem  Piperacillin/tazobactam | 6g (6g – 6g)  1g q6h (0.5g q6h – 1g q6h)  18g (18g – 18g) | No |
| Chua *et al*., 2022 | Meropenem,  Piperacillin/tazobactam | 3g (2g – 3g)  13.5g (9g – 13.5g) | No |
| Zhao *et al*., 2022 | Meropenem | Not reported | Yes |
| Alshaer *et al*., 2022 | Meropenem  Cefepime  Piperacillin/tazobactam | Not reported | No |
| Gatti *et al*., 2023 | Ceftazidime/avibactam | 2.5g q8h (CLCr > 50 mL/min/1.73m^2^)  1.25g q8h (CLCr 31-50 mL/min/1.73m^2^)  0.625g q8h (CLCr < 30 mL/min/1.73m^2^) | Yes |
| Alshaer *et al*., 2023 | Cefepime  Meropenem  Piperacillin/tazobactam | Not reported | No |
| Gatti *et al*., 2023 | Piperacillin/tazobactam | 18g (13.5g – 18g) | Yes |
| Udy *et al*., 2012 | Ampicillin  Dicloxacillin  Penicillin  Flucloxacillin  Piperacillin  Cephalothin  Cefazolin  Ceftriaxone  Ceftazidime  Cefepime  Meropenem  Ertapenem | Not reported | No |
| Hites *et al*., 2013 | Cefepime  Meropenem  Piperacillin/tazobactam | 2g q8h  1g q8h  4.5q 6h | No |
| Alobaid *et al*., 2016 | Piperacillin/tazobactam  Meropenem | 13.5g (9g – 18g)  1g q8h (0.5g q6h – 1g q8h) | No |
| Damen *et al*., 2019 | Piperacillin/tazobactam | 18g/day | No |
| Dhaese *et al*., 2019 | Piperacillin/tazobactam  Meropenem | 4.5g q6h  1g q8h | No |
| Fillatre *et al*., 2021 | Piperacillin/tazobactam | 4.5g q6h | No |
| Guilhaumou *et al*., 2023 | Cefepime  Cefotaxime  Ceftazidime  Meropenem | 4g/day  6g/day  6g/day  4g/day | Yes |
| Gatti *et al*., 2023 | Meropenem  Piperacillin/tazobactam  Ceftazidime/avibactam Meropenem/vaborbactam | 0.5g q6h (0.5g q6h – 1g q6h)  13.5g (9g – 18g)  1.25g q8h (1.25g q8h – 2.5g q8h)  2g/2g q8h (1g/1g q8h – 2g/2g q8h) | Yes |
| Tournayre *et al*., 2023 | Meropenem | 2g q8h or 1g q4h | No |

Data were expressed as range or median (interquartile range); CLCr: creatinine clearance.

**Supplementary Table 2 –** Risk of bias assessment for observational studies according to ROBINS-I tool

| **Study** | **Bias domains** | | | | | | | **Overall judgment** |
| --- | --- | --- | --- | --- | --- | --- | --- | --- |
|  | **Bias due to confounding** | **Bias in selection of participants** | **Bias in classification of exposure** | **Bias due to deviations from intended exposure** | **Bias due to missing data** | **Bias in measurement of outcome** | **Bias in selection of reported result** |  |
| Wong *et al*., 2018 | Moderate risk | Low risk | Low risk | Low risk | Low risk | Moderate risk | Moderate risk | Moderate risk |
| Carriè *et al*., 2018 | Moderate risk | Low risk | Low risk | Low risk | Low risk | Moderate risk | Moderate risk | Moderate risk |
| Abdulla *et al*., 2020  *(outcome: clinical cure)*  *(outcome:* *survival)* | Moderate risk  Moderate risk | Low risk  Low risk | Low risk  Low risk | Low risk  Low risk | Low risk  Low risk | Moderate risk  Low risk | Moderate risk  Moderate risk | Moderate risk  Moderate risk |
| Alshaer *et al*., 2020  *(both mortality and resistance occurrence)* | Moderate risk | Moderate risk | Low risk | Low risk | Low risk | Low risk | Moderate risk | Moderate risk |
| Taccone *et al*., 2021 | Moderate risk | Moderate risk | Low risk | Low risk | Low risk | Serious risk | Moderate risk | Serious risk |
| Gatti *et al*., 2021  (*both microbiological failure and resistance occurrence)* | Moderate risk | Moderate risk | Low risk | Low risk | Low risk | Moderate risk | Moderate risk | Moderate risk |
| Chua *et al*., 2022  *(outcome: clinical cure)*  *(outcome:* *survival)* | Moderate risk  Moderate risk | Low risk  Low risk | Low risk  Low risk | Low risk  Low risk | Low risk  Low risk | Moderate risk  Low risk | Moderate risk  Moderate risk | Moderate risk  Moderate risk |
| Zhao *et al*., 2022 | Moderate risk | Low risk | Low risk | Low risk | Low risk | Moderate risk | Moderate risk | Moderate risk |
| Alshaer *et al*., 2022  *(outcome: clinical cure)*  *(outcome:* *survival)* | Moderate risk  Moderate risk | Moderate risk  Moderate risk | Low risk  Low risk | Low risk  Low risk | Low risk  Low risk | Moderate risk  Low risk | Moderate risk  Moderate risk | Moderate risk  Moderate risk |
| Gatti *et al*., 2023 | Moderate risk | Moderate risk | Low risk | Low risk | Low risk | Low risk | Moderate risk | Moderate risk |
| Alshaer *et al*., 2023  *(outcome: clinical cure)*  *(outcome:* *mortality)* | Moderate risk  Moderate risk | Moderate risk  Moderate risk | Low risk  Low risk | Low risk  Low risk | Low risk  Low risk | Serious risk  Low risk | Moderate risk  Moderate risk | Serious risk  Moderate risk |
| Gatti *et al*., 2023  (*both microbiological failure and resistance occurrence)* | Moderate risk | Moderate risk | Low risk | Low risk | Low risk | Moderate risk | Moderate risk | Moderate risk |
| Udy *et al*., 2012 | Moderate risk | Moderate risk | Low risk | Low risk | Low risk | Low risk | Moderate risk | Moderate risk |
| Hites *et al*., 2013 | Moderate risk | Moderate risk | Low risk | Low risk | Low risk | Low risk | Moderate risk | Moderate risk |
| Alobaid *et al*., 2016 | Moderate risk | Moderate risk | Low risk | Low risk | Low risk | Low risk | Moderate risk | Moderate risk |
| Damen *et al*., 2019 | Moderate risk | Moderate risk | Low risk | Low risk | Low risk | Low risk | Moderate risk | Moderate risk |
| Dhaese *et al*., 2019 | Moderate risk | Low risk | Low risk | Low risk | Low risk | Low risk | Moderate risk | Moderate risk |
| Fillatre *et al*., 2021 | Moderate risk | Low risk | Low risk | Low risk | Low risk | Low risk | Moderate risk | Moderate risk |
| Guilhaumou *et al*., 2023 | Moderate risk | Low risk | Low risk | Low risk | Low risk | Low risk | Moderate risk | Moderate risk |
| Gatti *et al*., 2023 | Moderate risk | Moderate risk | Low risk | Low risk | Low risk | Low risk | Moderate risk | Moderate risk |
| Tournayre *et al*., 2023 | Moderate risk | Moderate risk | Low risk | Low risk | Low risk | Low risk | Moderate risk | Moderate risk |
