# Supplementary figures and images for: Impact of attaining aggressive vs. conservative PK/PD target on the clinical efficacy of beta-lactams for the treatment of Gram-negative infections in the critically ill patients: a systematic review and meta-analysis
Source: Crit Care. 2024 Apr 16;28:123. doi: 10.1186/s13054-024-04911-5 (PMC11020314; doi:10.1186/s13054-024-04911-5)

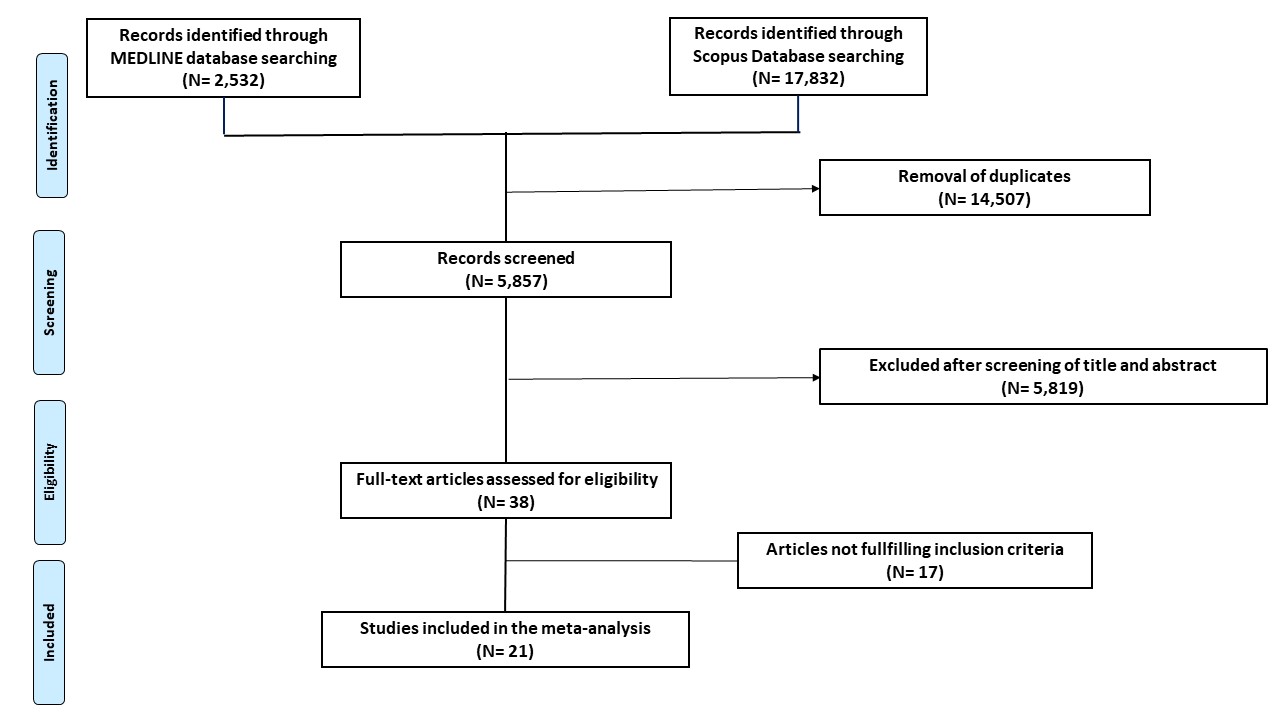

Supplement: Supplementary file 2 — Additional file 2. Supplementary Figure 1. PRISMA flow diagram for study selection. [file 13054_2024_4911_MOESM2_ESM.jpg]

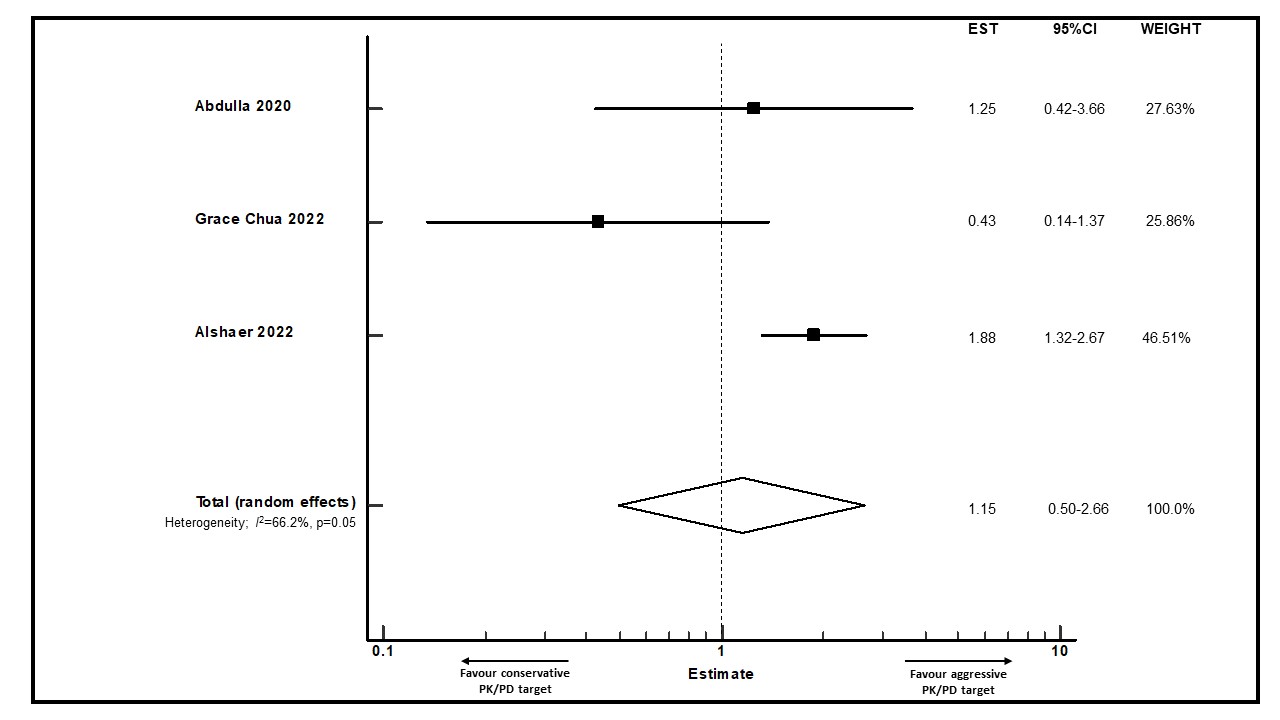

Supplement: Supplementary file 3 — Additional file 3. Supplementary Figure 2. Forest plot of survival rate in critically ill patients attaining aggressive vs. conservative beta-lactams PK/PD targets. [file 13054_2024_4911_MOESM3_ESM.jpg]

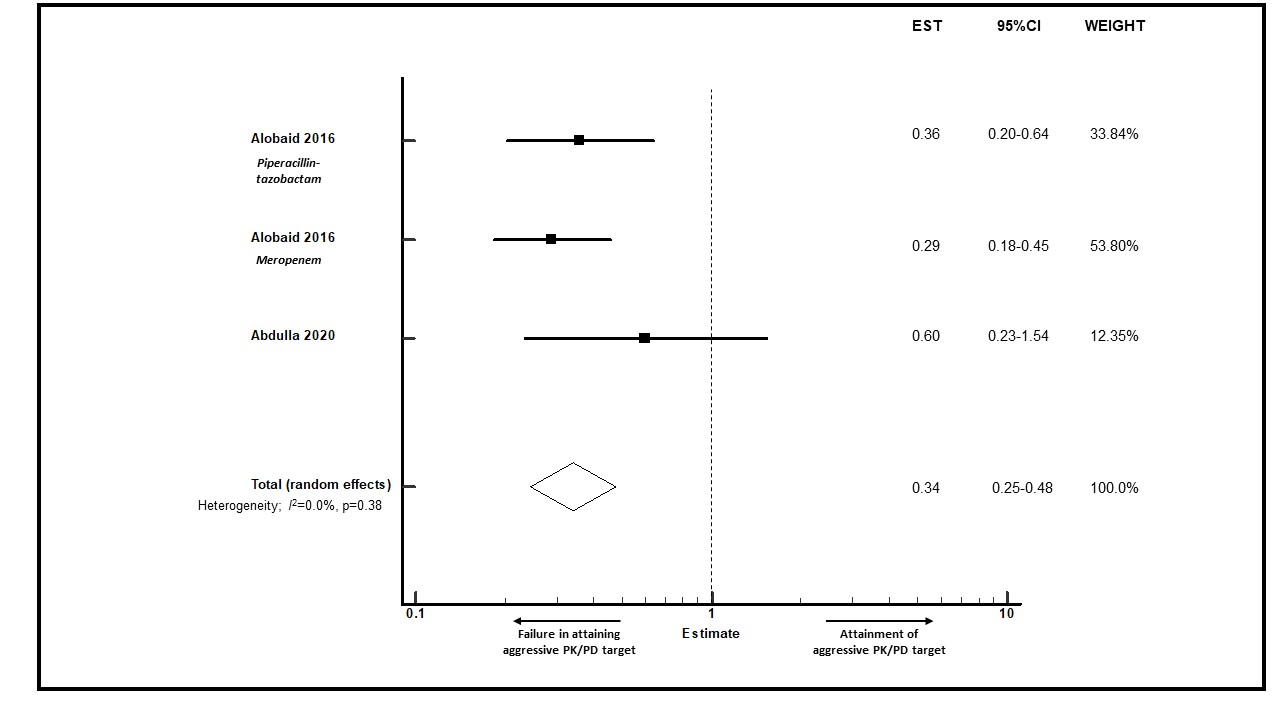

Supplement: Supplementary file 4 — Additional file 4. Supplementary Figure 3. Forest plot of the predictive factor male gender for failure in attaining aggressive beta-lactams PK/PD targets. [file 13054_2024_4911_MOESM4_ESM.jpg]

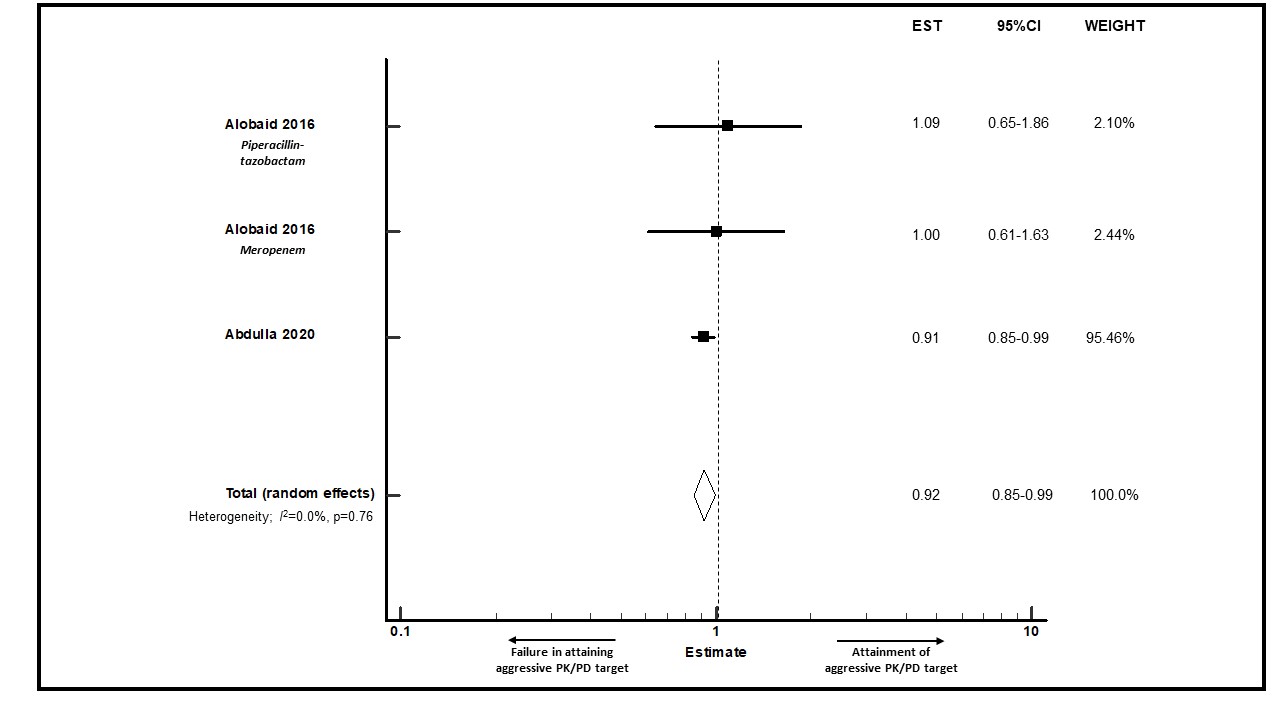

Supplement: Supplementary file 5 — Additional file 5. Supplementary Figure 4. Forest plot of the predictive factor BMI > 30 Kg/m2 for failure in attaining aggressive beta-lactams PK/PD targets. [file 13054_2024_4911_MOESM5_ESM.jpg]

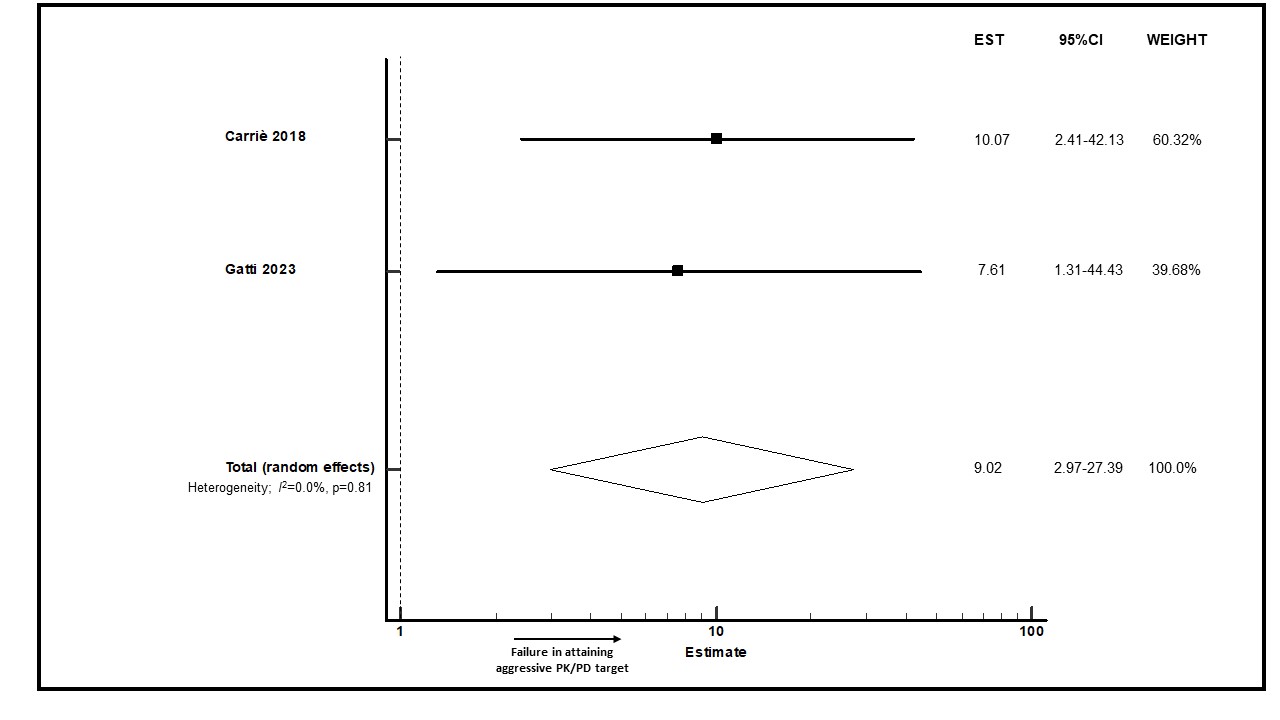

Supplement: Supplementary file 6 — Additional file 6. Supplementary Figure 5. Forest plot of the predictive factor ARC for failure in attaining aggressive beta-lactams PK/PD targets. [file 13054_2024_4911_MOESM6_ESM.jpg]

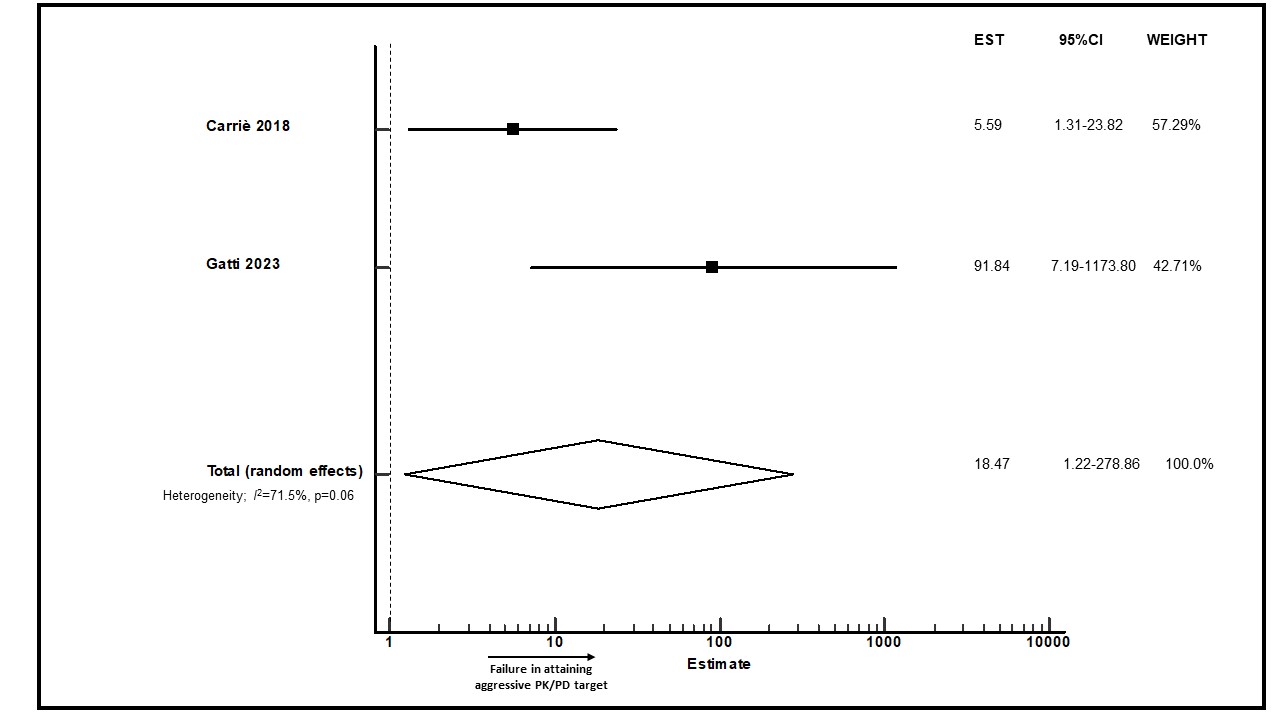

Supplement: Supplementary file 7 — Additional file 7. Supplementary Figure 6. Forest plot of the predictive factor MIC above clinical breakpoint for failure in attaining aggressive beta-lactams PK/PD targets. [file 13054_2024_4911_MOESM7_ESM.jpg]

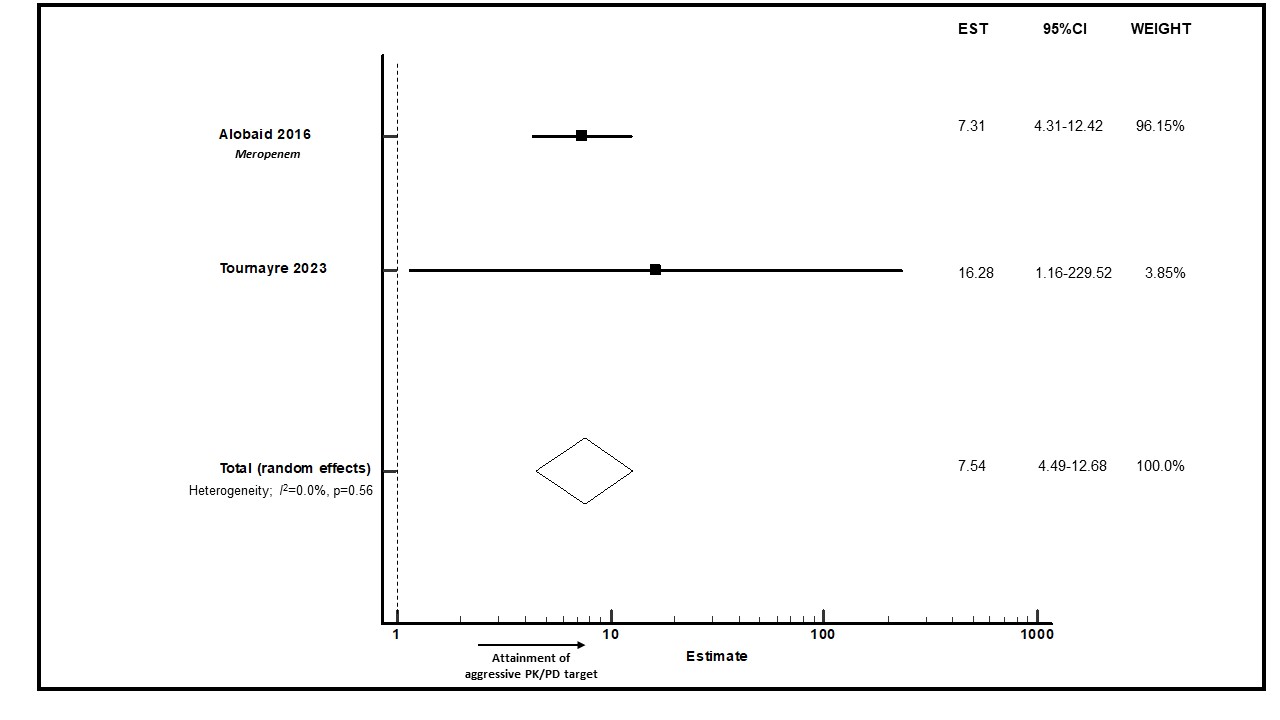

Supplement: Supplementary file 8 — Additional file 8. Supplementary Figure 7. Forest plot of the predictive factor prolonged infusion for attaining aggressive beta-lactams PK/PD targets. [file 13054_2024_4911_MOESM8_ESM.jpg]
